# Knowledge, experience and perception regarding molar incisor hypomineralisation (MIH) among dentists and dental hygienists in Oslo, Norway

**DOI:** 10.1007/s40368-021-00649-8

**Published:** 2021-08-12

**Authors:** A. B. Skaare, C. Houlihan, C. J. Nybø, I. J. Brusevold

**Affiliations:** Department of Paediatric Dentistry and Behavioural Science, Institute of Clinical Dentistry, Faculty of Dentistry, University of Oslo, Oslo, Norway

**Keywords:** Hypomineralisation, MIH, Children, Dental health personnel, Questionnaire

## Abstract

**Aim:**

The aim of this study is to gather baseline information on knowledge, perceptions, clinical experience and treatment options regarding MIH among dental care providers in Oslo, Norway, before a larger epidemiological study.

**Methods:**

An electronic questionnaire was distributed to dentists (*n* = 88) and dental hygienists (*n* = 47) working in the Public Dental Service (PDS) in Oslo. The questionnaire consisted of five sections related to sociodemographic, clinical experience, perceptions, clinical management and preferences for further training. Descriptive statistics with chi-squared test was used, and level of statistical significance was set to 5%.

**Results:**

Replies were obtained from 74.1% (*n* = 100) after two reminders. All respondents encountered MIH in their practice. The respondents’ perception of the prevalence of MIH in Oslo varied. The majority felt confident when diagnosing MIH (86%). The clinicians qualified in the last 10 years felt more confident than those who had qualified earlier (*p* = 0.016). Most were self-confident when treating these patients (68.3%), however, nearly all (88%) agreed that MIH was a clinical problem. The clinician’s treatment of MIH varied. Difficulties achieving adequate local anaesthetic (71.4%) and the child’s behavioural problems (84.1%) were treatment barriers for the dentists. Approximately two thirds (69%) would like further training, in particular on the aetiology (70%), diagnosis (57%) and treatment (77%) of the developmental disorder.

**Conclusion:**

All clinicians were familiar with the diagnosis of MIH and experienced the condition to be a clinical problem. Continuing education on aetiology, diagnosis and treatment of MIH is requested by dental health personnel.

## Introduction

Molar incisor hypomineralisation (MIH) is a highly prevalent condition. A worldwide estimate is 17.5 million new cases each year (Schwendicke et al. 2018) with a global prevalence ranging from 2.4 to 40.2% (Zhao et al. 2018). In Norway a prevalence of 13.9% is reported (Schmalfuss et al. 2016).

MIH is characterised by qualitative enamel defects in one or more of the first permanent molars (FPM), frequently associated with affected incisors. The diagnosis is achieved when a demarcated enamel hypomineralisation of systemic origin is registered in at least one first permanent molar (Weerheijm et al. 2001). The appearance of the tooth may be opaque with a white chalky, cream or yellow–brown colour. The enamel has normal thickness upon eruption and the surface may appear hard but it is prone to post-eruptive breakdown due to the hypomineralisation. The condition has an asymmetric presentation and often presents with marked variation in the severity of affected teeth in the same individual (Weerheijm 2004). Newer studies also describe similar defects in second primary molars and other permanent teeth (Negre-Barber et al. 2016; Schmalfuss et al. 2016; Elfrink et al. 2012; Mittal 2016; Kevrekidou et al., 2021). Although no definitive aetiological factor has been identified, several have been suggested. Environmental factors, chronic illness during last trimester of pregnancy and early childhood illnesses are those most consistently implicated (Lygidakis et al. 2008; Alaluusua 2010; Silva et al. 2016b).

Hypersensitivity, post-eruptive breakdown of enamel and the development of dental caries are clinically problematic (Weerheijm and Mejàre 2003; Elhennawy and Schwendicke 2016; Americano et al. 2017; Lygidakis et al. 2010). In addition, there is an aesthetic burden and MIH has been reported to negatively affect children’s general health, quality of life and psychosocial well-being. (Jälevik and Klingberg 2012; Lygidakis et al. 2010).

Obtaining adequate pain control, negotiating an optimal preparation border and selecting a reliable restorative material are some of the difficulties clinicians have reported when treating MIH-affected teeth (Crombie et al. 2008; Mejàre et al. 2005; William et al. 2006). Two surveys among Norwegian dentists working with children and adolescents have shown a notable disparity between clinicians’ views and on how to treat dental developmental defects like MIH (Kopperud et al. 2016; Uhlen et al. 2019). By the age of nine, children who have been diagnosed with MIH have had ten times more dental treatment as children without MIH, resulting in an increase in dental anxiety and behaviour problems (Jälevik and Klingberg 2012).

Studies on knowledge among dental clinicians have been conducted in other countries (Ghanim et al. 2011; Hussein et al. 2014; Silva et al. 2016a; Gambetta-Tessini et al. 2016; Gamboa et al. 2019) based on a survey by Crombie et al. (2008). In Norway, the number of specialist paediatric dentists are few and most paediatric dental care is provided by the general dental practitioners and the dental hygienists working in the Public Dental Service (PDS). The PDS accounts for one third of the total dental service in the country. All children under the age of 18 years are provided with free dental care at public clinics and the dental hygienists are often the first to identify children with MIH. Patients requiring restorative treatment are referred to the dentist. Treatment of MIH often requires both the child and parents to attend several dental appointments, thus the disease carries a heavy financial burden for both the individuals’ families and the state (Gambetta-Tessini et al. 2016).

Hence, the main aim of this study was to gather information about the current level of knowledge among the clinicians diagnosing and treating MIH patients. The objectives were to get baseline information on knowledge, perceptions, clinical experience and treatment options regarding MIH among dental care providers working in the PDS in Oslo before the onset of a large epidemiological study.

### Methods

In April 2017, an electronic questionnaire was sent to all oral health care professionals (*n* = 135) employed in the PDS in Oslo. The study was approved by the Norwegian centre for research data (NSD) (Project number: 51535). The questionnaire, modelled and with approval after that of Gambetta-Tessini et al. (2016), was translated to Norwegian and back translated to English by two independent translators to ensure the translations were without discrepancies. The chief dental officer provided the email addresses of 88 dentists and 47 dental hygienists. The questionnaire was distributed using a web-based software program (Questback Norway), ensuring the anonymity of the respondents. An information letter followed the questionnaire where it was stated that this survey was part of a planned epidemiological study among 8-years olds in Oslo on molar incisor hypomineralisation. It was further informed about anonymity, withdrawal and approval. Two reminders were sent automatically 2 weeks apart, followed by a final reminder in August 2017.

The questionnaire had five sections with a total of thirty-two pre-coded questions and five free text answers. The first section gathered background information on the respondents’ age, sex, profession, education and duration of practice (Table 1). Years in practice were dichotomized into ≤ 10 or > 10 years.

**Table 1:** Sociodemographic characteristics of dentists and dental hygienists

Characteristics	All (*n* = 100) *n* (%)	GDPs (*n* = 63) *n* (%)	Dental hygienists (*n* = 37) *n* (%)
Gender			
Female	91 (91)	54 (85.7)	37 (100)
Male	9 (9)	9 (14.3)	0 (0)
Age			
≤ 30	17 (17.0)	11 (17.4)	6 (16.2)
31–40	42 (42.0)	26 (41.3)	16 (43.3)
41–50	15 (15.0	10 (15.9)	5 (13.5)
≥ 51	26 (26.0)	16 (25.4)	10 (27.0)
Education			
Norway	83 (83.0)	48 (76.2)	35 (94.6)
Abroad	16 (16.0)	15 (23.8)	1 (2.7)
Missing	1 (1.0)	0 (0.0)	1 (2.7)
Years in practice			
≤ 5	20 (20.0)	15 (23.8)	5 (13.5)
6–10	30 (30.0)	17 (27.0)	13 (35.1)
11–20	30 (30.0)	14 (22.2)	16 (43.2)
21–30	12 (12.0)	12 (19.0)	0 (0.0)
≥ 31	8 (8.0)	5 (7.9)	3 (8.1)

**Fig. 1:**
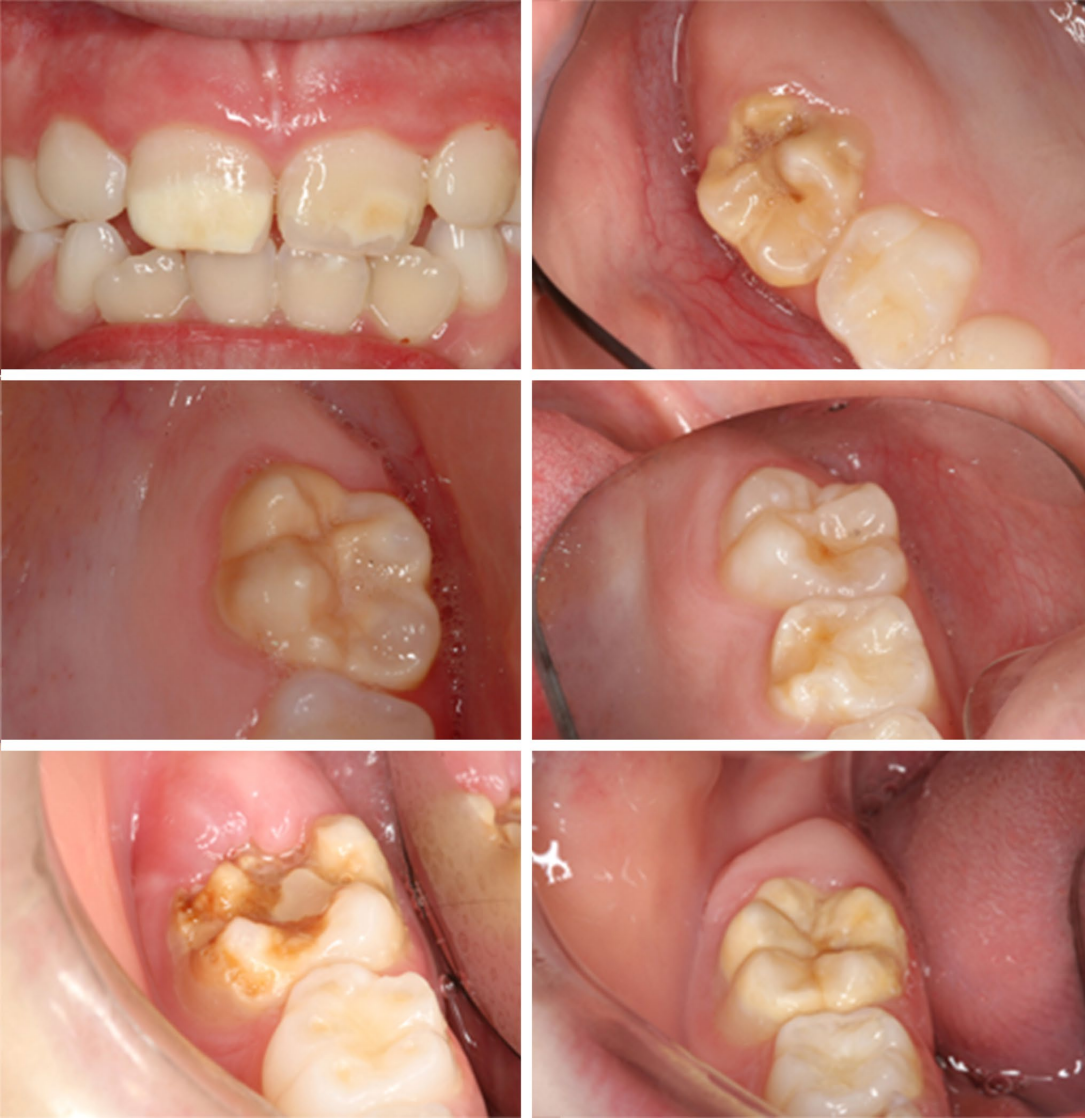
Photographs included in the questionnaire to illustrate different clinical manifestations of molar incisor hypomineralisation

The second part addressed their knowledge and clinical experiences regarding MIH (Tables 2 and 3) and was accompanied by nine close-up photographs illustrating different clinical manifestations of MIH, of which six are displayed in Fig. 1. In this section, the participants were asked if they encountered MIH in their practice, to specify the severity of the defects most frequently observed and if they observed defects on teeth other than first permanent molars and incisors. Questions on confidence in diagnosis, caries and other developmental defects as well as estimated prevalence and whether prevalence was worth surveying were also addressed.

**Table 2:** Knowledge and perception of molar incisor hypomineralisation among dentists and dental hygienists

Knowledge and perception		All *n* (%)	GDPs *n* (%)	Dental hygienists *n* (%)	*P* value
Do you encounter teeth with MIH in your practice?	Yes	100 (100)	63 (63.0)	37 (37.0)	
	No	0	0	0	
Regarding severity of the defect: which of the following do you most frequently notice in your practice?	White demarcated	43 (43.0)	26 (41.1)	17 (45.9)	0.740
	Yellow/brown demarcated	52 (52.0)	33 (52.3)	19 (51.3)	
	PEB	5 (5.0)	4 (6.3)	1 (2.7)	
In your practice, do you encounter demarcated hypomineralised defects on other permanent teeth than FPM and incisors?	Yes	74 (74.0)	44 (69.8)	30 (81.1)	0.216
	No	26 (26.0)	19 (30.1)	7 (18.9)	
How frequently do you notice this defect in the second primary molar compared to FPM and incisors?	More often	3 (3.0)	1 (1.6)	2 (5.4)	0.002*
	As often	9 (9.0)	1 (1.6)	8 (21.6)	
	More seldom	75 (75.0)	53 (84.1)	22 (59.5)	
	Not sure	13 (13.0)	8 (125)	5 (13.5)	
Do you feel the incidence of these defects has increased in the period of your practice?	Yes	51 (51.0)	30 (47.6)	21 (56.8)	0.059
	No	17 (17.0)	15 (23.8)	2 (5.4)	
	Not sure	32 (32.0)	18 (28.6)	14 (37.8)	
How confident do you feel when diagnosing teeth with MIH?	Very confident	7 (7.0)	4 (6.3)	3 (8.1)	0.816
	Confident	79 (79.0)	49 (77.8)	30 (81.1)	
	Unconfident	13 (13.0)	9 (14.3)	4 (10.8)	
	Very unconfident	1 (1)	1 (1.6)	0 (0)	
Do you think a significant percentage of caries is caused by MIH?	Yes	39 (39.0)	27 (42.8)	12 (32.4)	0.499
	No	44 (44.0)	25 (39.7)	19 (51.4)	
	Not sure	17 (17.0)	11 (17.5)	6 (16.2)	
Do you think the pattern (size, shape, location) of caries due to MIH is different from the “classical” caries pattern?	Yes	81 (81.0)	54 (85.7)	27 (73.0)	0.289
	No	6 (6.0)	3 (4.8)	3 (8.1)	
	Not sure	13 (13.0)	6 (9.5)	7 (18.9)	
Do you think MIH is a developmental defect of enamel that differs from dental fluorosis and hypoplasia?	Yes	87 (87.0)	57 (90.5)	30 (81.1)	0.177
	No	13 (3.0)	6 (9.5)	7 (18.9)	
How prevalent do you think MIH might be in your community?	< 5%	5 (5.0)	4 (6.3)	1 (2.7)	0.526
	5–10%	24 (24.0)	15 (23.8)	9 (24.3)	
	10–20%	31 (31.0)	20 (31.7)	11 (29.7)	
	20–30%	22 (22.0)	13 (20.6)	9 (24.3)	
	> 30%	4 (4.0)	4 (6.3)	0 (0)	
	Not sure	14 (14.0)	7 (11.1)	7 (18.9)	
Do you think it would be worthwhile investigating the prevalence?	Yes	95 (95.0)	59 (93.7)	36 (97.3)	0.419
	No	5 (5.0)	4 (6.3)	1 (2.7)	
Do you think teeth with MIH represent a clinical problem?	Yes	88 (88.0)	60 (95.2)	28 (75.7)	0.008*
	No	12 (12.0)	3 (4.7)	9 (24.3)	
If yes, how serious/severe do you think MIH is in your community?	Mild	11 (12.5)	7 (11.7)	4 (14.3)	0.877
	Moderate	52 (59.0)	37 (61.7)	15 (53.6)	
	Severe	19 (21.6)	12 (20.0)	7 (25.0)	
	Not sure	6 (6.8)	4 (6.7)	2 (7.1)	
Do you believe that early examinations are important to treat MIH?	Yes	88 (88)	59 (93.7)	29 (78.4)	0.041*
	No	4 (4)	2 (3.2)	2 (5.4)	
	Not sure	8 (8)	2 (3.2)	6 (16.2)	

^*^Statistically significant (*p* < 0.05). Fischer’s exact test when expected counts are less than five

**Table 3:** Confidence in diagnosing MIH according to years in practice

Years in practice	Confident	Not confident	Total
5 years or less	20	0	20
6–10 years	28	2	30
11–20 years	22	8	30
21–30 years	10	2	12
30 years or more	6	2	8
Total	88	14	100

^*^Statistical significant difference between age groups, *p* = 0.05

10 years or less in practice, *p* = 0.016

The third section consisted of two questions regarding aetiological factors and the period in which the causative insult had likely occurred (Table 4). The fourth section addressed only the dentists and included treatment options and difficulties commonly encountered when restoring MIH teeth. This included questions on whether they felt confident when treating children with MIH and whether they would refer a child with MIH to a specialist paediatric dentist (Table 5).

**Table 4:** Knowledge on aetiology of molar incisor hypomineralisation and time of insult (more than one option possible and only YES answers presented)

Aetiological factors	All *n* = 100 (%)	GDPs *n* = 63 (%)	Dental hygienists *n* = 37 (%)	*P* value
Genetics	71 (71.0)	41 (65.1)	30 (81.1)	0.089
Environmental contaminants	49 (49.0)	33 (52.4)	16 (43.2)	0.377
Chronic medical condition during pregnancy	40 (40.0)	24 (39.1)	16 (43.2)	0.612
Chronic medical condition of child	49 (49.0)	30 (47.6)	19 (51.4)	0.718
Acute medical condition during pregnancy	34 (34.0)	21 (33.3)	13 (35.1)	0.854
Acute medical condition of child	52 (52.0)	33 (52.4)	19 (51.4)	0.924
Antibiotics/medication taken by the mother during pregnancy	49 (49.0)	27 (42.9)	22 (59.5)	0.109
Antibiotics/medication taken by the child	61 (61.0)	36 (57.1)	25 (67.7)	0.302
Fluoride exposure or consumption	12 (12.0)	7 (11.1)	5 (13.5)	0.721
None of the above	0 (0.0)	0 (0.0)	0 (0.0)	
Not sure	22 (22.0)	19 (30.2)	3 (8.1)	0.01*
Other	11 (11.0)	9 (14.3)	2 (5.4)	0.205
During what time/period do you think this insult occurs?				
First trimester	14 (14.0)	9 (14.3)	5 (13.5)	0.914
Second trimester	22 (22.0)	13 (20.6)	9 (24.3)	0.667
Third trimester	36 (36.0)	23 (36.5)	13 (35.1)	0.890
First year of life	71 (71.0)	44 (69.8)	27 (73.0)	0.739
Second year of life	21 (21.0)	12 (19.0)	9 (24.3)	0.532
Third year of life	6 (6.0)	5 (7.9)	1 (2.7)	0.408
Not sure	18 (18.0)	14 (22.2)	4 (10.8)	0.185

^*^Statistically significant (*p* < 0.05). Fischer’s exact test when expected counts are less than five

**Table 5:** Treatment options (dentists only)

Management considerations		GDPs *n* = 63 (%)
Would you refer a child with MIH to a paediatric dentist for treatment?	Yes	3 (4.8)
	No	11 (17.4)
	Sometimes	49 (77.8)
What type of material do you most often use in the treatment of MIH?	Composite	49 (77.8)
	GIC	48 (76.2)
	RMGIC	9 (14.3)
	Stainless steel crowns	15 (23.8)
	Cast restoration	5 (7.9)
	Other	4 (6.3)
Do you feel confident when treating children with MIH?	Yes	43 (68.3)
	No	20 (31.7)
Would any of the following be a barrier to you for managing MIH teeth	Dental treatment that requires a long time to be accomplished	15 (23.8)
	Child behaviour (uncooperative child)	53 (84.1)
	Difficulty in achieving local anaesthesia	45 (71.4)
	Insufficient training to treat children with MIH	8 (12.7)
	Other	7 (11.1)

The final section addressed both dentists and dental hygienists on continuing education within the areas of diagnostics, aetiology and treatment (Table 6). Sections three to five included several options where the respondents were asked to select all applicable answers (Crombie et al. 2008; Ghanim et al. 2011; Hussein et al. 2014; Gambetta-Tessini et al. 2016).

**Table 6:** Continuing education (select all that apply)

Aspects of continuing education		All *n* = 100 (%)	GDPs *n* = 63 (%)	Dental hygienists *n* = 37 (%)	*p* value
Are you receiving information on MIH?	Yes	86 (86)	55 (87.3)	31 (83.8)	0.625
	No	14 (14)	8 (12.7)	6 (16.2)	
If yes, what is/are your source(s)?	Dental journals	64 (64)	41 (65.1)	23 (62.2)	0.971
	Continuing education	40 (40)	32 (50.8)	8 (21.6)	0.004*
	Brochures or pamphlets	19 (19)	7 (11.1)	12 (32.4)	0.005*
	The internet	31 (31)	19 (30.2)	12 (32.4)	0.699
	Books	24 (24)	19 (30.1)	5 (13.5)	0.068
	Other	25 (25)	13 (20.6)	12 (32.4)	0.139
Would you like clinical training regarding tooth hypomineralisation?	Yes	69 (69)	43 (68.3)	26 (70.3)	0.525
	No	17 (17)	12 (19.0)	5 (13.5)	
	Not answered	14 (14)	8 (12.7)	6 (16.2)	
If yes, in which area(s) would you like further training?	Diagnosis	57 (57)	33 (52.4)	24 (64.9)	0.223
	Aetiology	70 (70)	37 (58.7)	33 (89.2)	0.001*
	Treatment	77 (77)	53 (84.1)	24 (64.9)	0.027*

^*^Statistically significant (*p* < 0.05)

Data management was performed with IBM SPSS Statistics version 25.0 (Statistical Package for the Social Sciences; Chicago, IL, USA). Descriptive statistics and chi-squared tests, Fischer’s exact test when expected counts were less than five, were used for analysis and to compare groups. The level of statistical significance was set at *p* < 0.05.

## Results

Replies were received from 100 respondents (dentists = 63, dental hygienists = 37). A response rate of 74.6% was calculated. Respondents were mainly female (91%). The age ranged from 23 to 64 years (mean 41.5, SD ± 11). Our sample was found to be representative for dental clinicians employed in the PDS in Norway regarding both sex (national 81.9% female) and age (mean 41.5) (Statistics Norway, Dental Health). The majority of the respondents (83%) had received their dental education in Norway. The year of graduation varied from 1977 until 2016. The average number of years practicing was 13.5 (SD ± 10).

All respondents encountered MIH in their practice. Yellow/brown demarcated opacities were reported to be slightly more prevalent (52%) than white demarcated defects (43%) whereas post-eruptive breakdown was less frequently observed (5%). There was no significant difference between dentists and dental hygienists regarding severity of the defects diagnosed (Table 2). 74% of respondents reported that they have observed demarcated hypomineralised defects on permanent teeth other than FPMs and incisors. However, FPMs and incisors were often included in their comments leaving 46% Yes answers. Among permanent teeth other than FPMs and incisors, canines (27%), premolars (19%) and less often second molars (7%) were noted. On the specific question whether they observed these defects in second primary molars, three quarters of respondents answered scarcer than on FPMs.

The respondents’ perception of the prevalence of MIH in Oslo varied. 14% were not sure and a vast majority (95%) thought it would be worth investigating. Approximately half of respondents agreed that the prevalence of MIH appeared to have increased in their professional lifetime whereas just under a third were unsure about this (Table 2). Those who had qualified recently (≤ 10 years) felt more confident when diagnosing MIH than those who had qualified more than 10 years ago (*p* = 0.016, Table 3).

Most clinicians reported that MIH differed from the traditional caries pattern. Less than half thought that a significant amount of caries was due to MIH. The majority agreed that MIH differed clinically from other developmental defects in enamel such as dental fluorosis or hypoplasia. Significantly more dentists than dental hygienists reported that MIH was a clinical problem (*p* = 0.008), and the majority of respondents agreed that early examination was important in identifying patients with MIH, more dentists than dental hygienists were of this opinion (*p* = 0.041) (Table 2).

The respondents suggested a variety of views with regards to possible aetiological factors and the period of the aetiological insult (Table 4). The majority blamed genetic factors and medication/antibiotic use during childhood. Approximately half reported acute or chronic childhood illness as causative factors. Many respondents believed that acute or chronic maternal illness and medication/antibiotic use during pregnancy were aetiological factors. More dental hygienists were uncertain (*p* = 0.01). Most respondents suspected that the aetiological insult for MIH occurred in the first year of life or during the third trimester of pregnancy, although quite a few also reported first and second trimester of pregnancy (Table 4).

Table 5 illustrates the dentists’ treatment options. The majority would occasionally refer a child with MIH to a specialist paediatric dentist for treatment, a few regularly. Composite resin and glass-ionomer were the most commonly used filling materials. Three quarters of dentists would use these materials on a regular basis while stainless steel crowns were reported by approximately one fourth of the practitioners. Resin-modified glass-ionomer cements and cast restorations were less frequently used. Most dentists (68.3%) felt confident when treating patients with MIH. However, a large majority reported difficulties achieving adequate local anaesthetic (71.4%), as well as the child’s behavioural problems (84.1%) as common barriers for treating MIH.

Table 6 shows how the dentists and dental hygienists received information on MIH. A common source for both groups were dental journals (64%). Courses/continuing education were more often reported by dentists than dental hygienists (*p* = 0.004). Brochures or pamphlets were less used, but were more common by dental hygienists (*p* = 0.005). The internet was a source used by both groups. Just over two thirds of dentists and dental hygienists would like further clinical training regarding tooth hypomineralisation, and more than half on diagnosis. Three quarters of the respondents wanted more training on aetiology and treatment, more dental hygienists on aetiology and more dentists on treatment (Table 6).

## Discussion

This is the first study to investigate the knowledge, perceptions, clinical experience and treatment options regarding MIH among dentists and dental hygienists in Norway. Thus, little is known on dental clinicians’ views on MIH’s frequency, diagnosis and treatment. Such knowledge is essential for the Public Dental Service (PDS) when planning strategies to improve oral health for children as the PDS in Norway is the main provider of children’s dental services and the sole provider of free dental care for children (0–18 years) in the country. Thus, nearly all children (97.6%, Statistics Norway, Dental Health, Statistics Norway, 2017) were enrolled in the PDS and regularly screened. Dentists and dental hygienist included in this study were working in the PDS in Oslo, the capital city of Norway. The respondents thus see a much larger proportion of children than a general dentist working in private practice. A strength of the study is the high response rate and there is no reason to believe that the respondents differ from other dental care providers working elsewhere in the PDS in Norway.

All the respondents in this survey had seen patients with MIH in their practice. This differs somewhat from similar studies (Ghanim et al. 2011; Hussein et al. 2014; Crombie et al. 2008; Gambetta-Tessini et al. 2016; Gamboa et al., 2018), but can be explained by the fact that the children are frequently seen before they develop caries or receive restorations masking a possible defect. The most frequently observed defect was yellow/brown opacities. The respondents´ experiences are thus similar to those reported in other international surveys (Ghanim et al. 2011; Hussein et al. 2014; Crombie et al. 2008; Gambetta-Tessini et al. 2016). Ghanim et al. (2011) have suggested that this may be because these opacities are easier to differentiate from alternative diagnosis such as dental fluorosis or white spot lesions. The more severe defect, post-eruptive breakdown, was least frequently observed which is in line with findings by Jälevik et al. (2001) reporting that 6.4% of the children had such defects. The majority of respondents reported that MIH differed clinically to other dental development disturbances, such as fluorosis and hypoplasia, suggesting a degree of confidence in their ability to diagnose MIH. Whether MIH is a precise term for the condition may be discussed. Mittal (2016) concluded that enamel hypomineralisation can manifest in any tooth in five different phenotypic variations, and in the newly published study by Kevrekidou et al. (2021), 22.9% of adolescents were diagnosed with hypomineralisation of permanent teeth other than FPMs and incisors. A strong association with MIH was observed as children with MIH had an odds ratio of three to present hypomineralisation in other teeth. In the present study, approximately half of the respondents had observed demarcated hypomineralised defects in other teeth than FPMs and incisors. This is in accordance with previous findings (Hussein et al. 2014; Gambetta-Tessini et al. 2016; Gamboa et al., 2018). Moreover, a similar prevalence was reported in a Norwegian study among 16-year-olds (Schmalfuss et al. 2016) where 23% of the subjects had affected canines, compared to the present estimation of 27%.

The participants in this survey were uncertain about the prevalence of MIH in their community with a slight majority estimating it to be 10–20%. This uncertainty was also a common finding in other international surveys (Ghanim et al. 2011; Weerheijm and Mejàre 2003; Hussein et al. 2014; Gambetta-Tessini et al. 2016). The vast majority of respondents thought this would be worth investigating, as to date there has only been one prevalence study conducted in Norway (Schmalfuss et al. 2016). Approximately half of respondents had the opinion that the prevalence of MIH appears to be increasing. Going to the literature this shows some diversity, varying from 15–20% reported by dental clinicians in Hong-Kong and Saudi-Arabia (Gamboa et al., 2018; Silva et al. 2016a), 38% among Iraqi dental academics (Ghanim et al. 2011) to approxiamtely 80% reported in Australia and Spain (Gambetta-Tessini et al. 2016; Serna-Muñoz et al. 2020). The only finding of a real increase and not only due to an enhanced awareness of MIH in recent years, are the reports from Australian clinicians in 2008 and 2016 (Crombie et al. 2008; Gambetta-Tessini et al. 2016).

The increased caries rate recorded in MIH patients (Kühnisch et al. 2018; Leppaniemi et al. 2001; Ghanim et al. 2013) was also evident in the present study, as 39% of respondents reported that a significant number of carious lesions was due to MIH. MIH teeth are often hypersensitive which can result in poor tooth brushing, plaque accumulation and rapid caries progression (Leppaniemi et al. 2001). The affected teeth are hypersensitive due to changes in pulpal innervation and inflammation and thus sensitivity to thermal changes (Rodd et al. 2007). According to dental health statistics, Norway has a low caries prevalence. 60% of 12-year-old children has DMFT = 0, thus making the diagnosis of caries as a result of MIH easier to identify in this population. Many respondents also reported the presence of atypical carious lesions. This has also been described as a common finding in a study among oral health care practitioners in Chile and Australia (Gambetta-Tessini et al. 2016) and is likely due to post-eruptive breakdown.

The variety of opinions regarding the aetiology of MIH supports the likely multifactorial nature of the condition. Surveys of dental communities in Iraq, Malaysia, Australia and New Zealand have also resulted in a variety of putative aetiological factors (Ghanim et al. 2011; Hussein et al. 2014; Crombie et al. 2008). In this survey, the majority suspected genetic factors had a role to play which may be a result of a family history among their affected patients rather than being updated on recent international publications. Jeremias et al. (2013) identified that genetics is an important aetiological factor and more recently it has been reported that genes modulating immune responses may have a synergistic effect, thus increasing the odds of an individual developing MIH (Bussaneli et al. 2018). Medication and antibiotic treatment as well as maternal illness during pregnancy were considered to be important. Many respondents also suspected that the aetiological insult occurred during the first and second trimester of pregnancy. Although the second primary molars mineralise during the second and third trimester of pregnancy, this shows deficient knowledge as mineralisation of the first permanent molars (mandatory for the MIH diagnosis), starts at birth. In addition, a systematic review by Silva et al. (2016b) failed to find an association between MIH and maternal illness or medication use in pregnancy. They reported that there was only weak evidence for birth complications and contradictory findings linking prematurity to MIH. However, early childhood illnesses, in particular those presenting with fever were implicated as etiological factors in several studies (Silva et al. 2016b) which supports the genetic-immune modulation theory outlined above (Bussaneli et al. 2018).

The management of MIH is challenging as the severity can vary greatly and the treatment must be individualised and adopted according to the patient’s needs. In this survey, MIH was reported as a clinical problem for more dentists than dental hygienists. This is not surprising given that dental hygienists do not provide restorative treatment. The dentists reported particular difficulties in obtaining local anaesthesia and managing the child’s behaviour. The failure to provide adequate analgesia may be explained by both innervation density in the pulp chamber and inflammatory reactions due to bacteria in the dentinal tubules (Rodd et al 2007; Fagrell et al. 2008). This information is important as painful treatment experiences can be the cause of dental anxiety (Skaret et al. 1999) and stress among the dentists performing restorative treatment in young patients (Rønneberg et al. 2015). Furthermore, a higher rate of behavioural management problems and difficulty with anaesthesia in MIH patients have been reported in Sweden (Jälevik and Klingberg 2012).

The most popular restorative materials used were glass-ionomer and composite resin. A survey of members of the Australian and New Zealand Society of Paediatric Dentistry reported similar practices (Crombie et al. 2008). Elhennawy and Schwendicke (2016) completed a systematic review on the management of MIH. They found an estimated mean annual failure rate of 12% for glass-ionomer restorations, a 4% annual failure rate for composite restorations and a 1.3% annual failure rate for stainless steel crowns. In the current survey, almost a quarter of the dentists reported the use of stainless steel crowns when treating MIH as recommended in the EAPD guidelines for the treatment of moderate to severely affected MIH teeth (Lygidakis et al. 2010). Stainless steel crowns are a treatment modality that has traditionally been more commonly offered by specialist paediatric dentists and their use among general dentists in the public sector is reported to be rare (Tran and Messer 2003; McKnight-Hanes et al. 1991). The common use reported in this survey may indicate a shift in modern day treatment practices or it may be that respondents have simply over-reported their use of stainless steel crowns. The latter is supported by a recent publication on the use of stainless steel crowns in Norway and Finland showing that this was an infrequent treatment choice among general dentists when restoring young molars (Uhlen et al. 2021). Elhennawy and Schwendicke (2016) reported that both direct and indirect restorations were a reliable treatment options with a very low annual failure rate. However, indirect restorations were not a commonly chosen treatment option among our respondents, which may be due to the high laboratory costs associated with this treatment.

In cases of severe MIH, extraction should be considered for FPMs of poor prognosis (Elhennawy and Schwendicke 2016). Extraction of the FPM may be the best treatment option provided it is planned well (Ashley and Noar 2019; Lygidakis et al. 2010). Tooth removal prevents the need for further interventions and may relieve the burden of regular dental procedures in the young patient. Extraction of FPMs was not a treatment option offered in this survey. The decision to extract or not can be a difficult one to make and a comprehensive evaluation of the compromised FPMs should be performed before planning an extraction (Willmot et al. 2008; Saber al. 2018). This challenging evaluation is likely to be the reason there is a high referral rate of MIH patients to specialist paediatric dentists reported in this survey. A similarly high referral rate was reported by Hussein et al. (2014), reporting that almost 60% of dentists would refer a child with MIH to a specialist.

The last part of the survey aimed to investigate the respondents’ continued professional development practices. It was encouraging that the majority of dentists and dental hygienists had received continuing education from dental journals and courses, which are likely to be reliable and validated sources of information. Considering the large number of respondents who would like further training in aetiology, diagnostics and treatment, formalised continuing education programs on MIH might increase confidence and knowledge among dental professionals working in the PDS.

A limitation of the study is the self-reported nature of the questionnaire which may increase response bias. The sample size is small with few male respondents and including both dentists and dental hygienists. However, the population is representative for these professions in the PDS in Norway. Nevertheless, the present study provides baseline information for the dental service.

## Conclusion

Within the limitations of the present study it was evident that molar incisor hypomineralisation is a prevalent condition encountered by all dentists and dental hygienists working in the Public Dental Service in Oslo. The respondents were uncertain about the prevalence of MIH in their community and the vast majority thought it would be worth investigating. A variety of views regarding possible aetiological factors highlights the multifactorial nature of the condition, but also indicates the need for additional theoretical education. Nearly all clinicians experienced MIH to be a serious clinical problem, while anaesthesia and behavioural management appeared the main treatment barriers. Further research with subsequent clinical training on MIH is warranted.
